# Draft Genome Sequences of Two Basidiomycetous Yeasts, Tremella yokohamensis and Tremella fuciformis

**DOI:** 10.1128/mra.00573-22

**Published:** 2022-09-22

**Authors:** Kazuhiro Chiku, Shiori Sugiyama, Kona Akimoto, Mitsuru Yoshida

**Affiliations:** a Faculty of Applied Life Science, Nippon Veterinary and Life Science University, Musashino-shi, Tokyo, Japan; University of Maryland School of Medicine

## Abstract

In this study, the genome sequences of two Basidiomycetous yeasts, Tremella yokohamensis and Tremella fuciformis, which have very similar morphological characteristics, were determined. The genomic sequence data obtained will be useful for understanding the taxonomy and metabolic-related genes of basidiomycete yeasts.

## ANNOUNCEMENT

Tremella yokohamensis strain NBRC100148 was isolated from the dead trunk of a *Quercus* sp. tree in Chiba Prefecture, Japan, in 2012. It was initially named Tremella fuciformis based on its morphological characteristics but was subsequently reclassified as Tremella yokohamensis in 2019 by phylogenetic analysis based on the D1/D2 domain of the 26S nuclear rRNA gene and the internal transcribed spacer regions showing it to be a close relative of the Tremella fuciformis clade (1). In this study, we analyzed the draft genome sequences of *T. yokohamensis* and *T. fuciformis* to compare their genome sequences.

*T. yokohamensis* strain NBRC100148 and *T. fuciformis* strain NBRC9317 were obtained from the Biological Resource Center, NITE (Tokyo, Japan). Each strain was precultured on MYS agar medium containing 1% (wt/vol) malt extract, 0.4% yeast extract, 1% sucrose, and 1.5% agar at 25°C and main-cultured in YMS liquid medium (without agar) at 25°C for 2 days with shaking at 150 rpm. Genomic DNA was extracted using the ISOPLANT II kit (NIPPON Gene, Tokyo, Japan). The genomes were analyzed via shotgun sequencing. The genomes were fragmented randomly by ultrasonication, and a full-length sequencing library for Illumina sequencing was prepared using the NEBNext DNA library prep kit (New England Biolabs, Ipswich, MA, USA). The libraries were purified using AMPure XP beads (Beckman Coulter, Brea, CA, USA) after PCR amplification. Whole-genome sequencing was performed using the Illumina NovaSeq 6000 sequencing system (Santa Clara, CA, USA). Default parameters were used for all software unless otherwise specified. The reads obtained were filtered to remove adapter, poly(A/T), and poly(G) sequences and low-quality and short (<100-bp) reads using Trimmomatic v.0.39 (2). The quality of the reads was checked using FastQC v.0.11.9 (http://www.bioinformatics.babraham.ac.uk/projects/fastqc/). *De novo* genome assembly was performed using Plantanus-allee v.2.2.2 (3). QUAST v.5.0.2 (4) was used to determine the statistics of the assembled genome (Table 1). For *T. yokohamensis* NBRC100148, we obtained 49,067,935 paired-end reads (2 × 150 bp), 328 contigs with an *N*_50_ value of 207,856 bp, and a GC content of 56.3% from 48,320,431 filtered subreads and 67 scaffolds. A total of 20,989,627 bp were assembled. For *T. fuciformis* NBRC9317, we obtained 44,222,087 paired-end reads (2 × 150 bp), 1,063 contigs with an *N*_50_ value of 58,412 bp, and a GC content of 56.5% from 43,436,583 filtered subreads and 311 scaffolds. A total of 22,427,922 bp were assembled. The genome size was similar to the results obtained for *T. fuciformis* strain tr26 (GenBank assembly accession number GCA_000987905). The genome sequences of the genus *Tremella* obtained in this study are extremely important for understanding the classification and phylogenetic evolution of basidiomycete yeasts and their ability to produce biologically active substances.

**TABLE 1:** General features of the *Tremella yokohamensis* and *T. fuciformis* genomes

Feature	Data for strain:	
	*Tremella yokohamensis* NBRC100148	*Tremella fuciformis* NBRC9317
No. of paired-end reads	49,067,935	44,222,087
Genome size (Mb)	21.0	22.4
GC content (%)	56.3	56.5
Genome coverage (×)	704	600
No. of contigs	328	1,063
Contig *N*_50_ (bp)	207,856	58,412
Contig *L*_50_ (bp)	27	240
No. of scaffolds	67	311
Scaffold *N*_50_ (bp)	1,154,394	224,639
DDBJ assembly accession no.	BRDC01000001 to BRDC01000067	BRDD01000001 to BRDD01000311
BioSample accession no.	SAMD00467664	SAMD00467665
BioProject accession no.	PRJDB13453	PRJDB13453

### Data availability.

The genome sequences of *T. yokohamensis* strain NBRC100148 and *T. fuciformis* strain NBRC9317 were deposited as whole-genome shotgun sequencing projects at the DDBJ/EMBL/GenBank under accession numbers BRDC00000000 and BRDD00000000. The versions described in this paper are the first versions, BRDC01000000 and BRDD01000000. The raw data have been deposited in the DDBJ Sequence Read Archive under accession numbers DRR374241 and DRR374242.

## References

[1] Malysheva VF, Malysheva EF, Bulakh EM. 2015. The genus Tremella (Tremellales, Basidiomycota) in Russia with description of two new species and proposal of one nomenclatural combination. Phytotaxa 238:40. doi:10.11646/phytotaxa.238.1.2.

[2] Bolger AM, Lohse M, Usadel B. 2014. Trimmomatic: a flexible trimmer for Illumina sequence data. Bioinformatics 30:2114–2120. doi:10.1093/bioinformatics/btu170. 24695404 PMC4103590

[3] Kajitani R, Yoshimura D, Okuno M, Minakuchi Y, Kagoshima H, Fujiyama A, Kubokawa K, Kohara Y, Toyoda A, Itoh T. 2019. Platanus-allee is a de novo haplotype assembler enabling a comprehensive access to divergent heterozygous regions. Nat Commun 10:1702. doi:10.1038/s41467-019-09575-2. 30979905 PMC6461651

[4] Gurevich A, Saveliev V, Vyahhi N, Tesler G. 2013. QUAST: quality assessment tool for genome assemblies. Bioinformatics 29:1072–1075. doi:10.1093/bioinformatics/btt086. 23422339 PMC3624806

